# m5C methylation modification guides the prognostic value and immune landscapes in acute myeloid leukemia

**DOI:** 10.18632/aging.205059

**Published:** 2023-09-25

**Authors:** Ya Liu, Yiying Chen, Maoping Cai, Yunguang Hong, Xiang Wu, Songyu Li

**Affiliations:** 1 Zhanjiang Institute of Clinical Medicine, Zhanjiang Central Hospital, Guangdong Medical University, Zhanjiang 524045, China; 2 Institute of Basic Medical Sciences Chinese Academy of Medical Sciences, School of Basic Medicine Peking Union Medical College, Beijing 100005, China

**Keywords:** acute myeloid leukemia, m5C, tumor microenvironment, immunotherapy

## Abstract

The development, incidence, and metastasis of tumors are all intimately correlated with 5-methylcytosine (m5C). However, uncertainty surrounds the function of m5C in acute myeloid leukemia (AML). In this study, multicenter AML data were collected and analyzed comprehensively to grasp the gene expression level, clinicopathological characteristics, prognostic significance of m5C in AML and its relationship with the tumor microenvironment (TME). The m5C gene-mediated scoring system (m5CSS) was created using principal component analysis, and multiple cox regression analyses were utilized to determine the prognostic relevance of the m5C score. The investigation of the correlation among m5C, immune characteristics, clinical characteristics, immune infiltration level, as well as drug reaction at immune checkpoints, and immunotherapy efficacy confirmed that the change of the characteristics of immune cell infiltration and patient prognosis are linked with the m5C gene. Moreover, the m5CSS was employed to assess the pattern of m5C modification. Further analyses showed that the m5C score can served as a reliable indicator of AML prognosis. Crucially, the prognostic value of the m5C score was validated in terms of drug resistance and immunotherapy. This work reveals that AML diversity and the generation of complex TMEs are both impacted by m5C modifications. Therefore, understanding the m5C modification pattern will improve grasp of TME infiltration characteristics and assist exploring more efficient immunotherapeutic approaches.

## INTRODUCTION

AML is a heterogeneous hematologic cancer distinguished by the growth of progranulocytes or bone marrow primitive cells that cannot properly develop [1]. In the United States, there were a projected 21,450 new cases of AML in 2019, which accounted for 1.2% of all new cancer diagnoses. The incidence rises with age, with instances hitting people between the ages of 65 and 74 at a rate of 25.1% and those over 75 at a rate of 33.7%. In addition, AML patients have a 28.3 % five-year survival rate, and there will likely be 10,920 AML fatalities in 2019. Similarly, the rate of both mortality and morbidity increases with age, and is highest in individuals at the age of 75 or older (43.7%) [2]. Despite the development of cellular and immunotherapies, chemotherapies, and targeted therapies [3, 4] that have provided more options of treatment modalities for patients, AML is still a type of disease which is complex and difficult to treat. Awfully, the development of reliable biomarkers for precision medicine remains a challenge.

A possible target for therapy is aberrant RNA alterations since they are linked to cancer cell survival, proliferation, invasion, and resistance to treatment [5, 6]. RNA species such as messenger RNA (mRNA), ribosomal RNA (rRNA), transporter RNA (tRNA), and non-coding RNA (ncRNA) are all commonly found in bacteria, prokaryotes, and eukaryotes. High-throughput and biochemical studies have demonstrated that m5C is a common RNA modification in these RNA species [7–11]. So far, 95,391 m5C sites in the human genome have been identified [12]. It is a typical human RNA alteration that has a significant impact on RNA biology [9, 13]. By changing the conformation of rRNA to control ribosome production and processing, m5C has an impact on translation. m5C sites on tRNA are evolutionarily conserved and help maintain tertiary structure, and it also functions in mRNA, affecting its transport and translation [14–16]. Recent researches have revealed that m5C can efficiently increase gene expression levels and decrease a few tumor suppressor genes via promoter hypermethylation [17, 18]. Importantly, expression levels of genes controlled by m5C are related to tumor development [19–23].

It has been demonstrated in numerous studies that another important element in the growth of tumors is the microenvironment in which tumor cells thrive and develop. The immune system, cancer cells, and extracellular matrix make up the heterogeneous system known as the tumor microenvironment (TME) [24, 25]. With increasing knowledge concerning variety and complexity of the TME, more and more evidence support its significance for tumor development, immune escape, and the effectiveness of immunotherapeutic treatment. TME affects cancer onset and progression, correlating with clinicopathological features such as age, grade, stage, and molecular subtypes with prognosis [26, 27]. Accordingly, it is conceivable to identify different tumor immunophenotypes and look for interesting biomarkers by thoroughly addressing the heterogeneity and complexity of TME [28].

In our study, we systematically described the expression of the m5C gene in AML. The association among m5C and clinical factors, immune infiltration levels, checkpoint drug response, and immunotherapy efficacy were also analyzed. It confirmed that multilayer alterations of the m5C gene were connected to patients’ prognosis and immune cell infiltration characteristics. Additionally, a m5C gene-mediated scoring system (m5CSS) was constructed to quantify these subtypes in AML by m5C score, which provided a feasible reference for the optimization of therapeutic regimens in AML.

## MATERIALS AND METHODS

### AML data set collection

Clinical data (Table 1) was obtained using the R program cgdsr, and the mRNA expression profile data and sample copy number variation (CNV) information for AML were collected from the UCSC Xena database (https://xenabrowser.net/datapages/). The R package “TCGAbiolinks” was utilized to obtain somatic mutation data. The GEO database (https://www.ncbi.nlm.nih.gov/geo/) was used to retrieve expression data and matched survival data of the microarray dataset GSE37642 as the validation set. Patients without a record of survival were disqualified. The collection of 21 m5C genes used to identify different cell types was taken from a study by Jiao Hu et al. [29].

**Table 1:** Clinical information statistics of TCGA-LAML.

**Characteristic**	**Number of patients**
**Age**	
<=65	86
>65	31
**Sex**	
Female	52
Male	65
**FAB**	
M0	11
M1	28
M2	29
M3	11
M4	25
M5	10
M6	2
M7	1

### Gene set variation analysis

The most common application of the unsupervised, non-parametric Gene Set Variation Analysis (GSVA) technique is to estimate changes in the pathway and biological process activity of samples. We performed GSVA enrichment analysis using the R package GSVA to examine the variation in expression levels of distinct sample types in relation to biological processes. The Molecular Signature Database (MSigDB; https://www.gsea-msigdb.org/gsea/msigdb/) was used to acquire the gene set h.all.v7.4.symbols.gmt of the GSVA. Statistical significance was defined as an adjusted P-value< 0.05.

### TME cell immune infiltration assessment

The Wilcoxon test was used to examine the distribution of immune cell infiltrations in various subgroups of samples in order to assess the TME cell immune infiltration. The percentage of infiltrations was estimated based on the following three modalities. (1) The single-sample gene set enrichment analysis (ssGSEA) was originally used to express the relative abundance of each cell infiltrate in TME. The gene set labeling TME-infiltrating immune cell type came from a study by Pornpimol Charoentong et al. [30]. It contained 28 different varieties of human immune cells, such as dendritic cells, activated CD8 T cells, macrophages, etc. (2) The 22 cell phenotype proportions in the samples were calculated using CIBERSORT combining LM22 feature matrix, with predicted immune cell percentage of each sample added together equaling 1. (3) The method from the xCell R package was also applied to compute the percentage of 64 immune cell infiltrations. Finally, the ESTIMATE method was applied to calculate the immune score, stromal score, and tumor purity of each tumor sample. The differences in immunological score, stroma score, and tumor purity were then compared between the sample groups by using Wilcoxon test.

### Unsupervised clustering of m5C-related regulators

In order to ensure the stability of the classification and to determine the survival of samples with various subtypes, the ConsensusClusterPlus package for consistent clustering analysis with 1000 replications was employed to disease type cancer samples using the expression of m5C-related genes. Based on the subtypes obtained, genes significantly differentially expressed (|log2FC|>1 and adjP<0.05) were analyzed using limma. To search for significantly enriched biological processes and associated pathways, differential genes were subjected to GO and KEGG enrichment analyses (P<0.05). The consistency clustering analysis for differential expressed genes was also performed using the ConsensusClusterPlus package to parse the survival of various categories.

### Construction and validation of m5C scoring system (m5CSS)

Combined with differential genes, the protein-coding genes among them were screened. To identify genes strongly connected to OS, univariable cox regression analysis was used (P < 0.005). m5CSS was constructed based on prognosis-related genes using principal component analysis (PCA). The calculation formula was as follows:


$$m5CSS=\sum \left(PC1_{i}+PC2_{i}\right)$$

The m5C score was produced in the validation set using the same formula. For the high and low group samples, the median value was chosen as the threshold for categorization. The predictive ability of the scoring system was evaluated using ROC curves and Kaplan-Meier survival analyses. According to the categories to which the above samples belonged, the CNV data from several groups of samples were tested for significant amplification deletion levels using the GISTIC2 tool from the GenePattern website (https://www.genepattern.org/).

### Drug sensitivity and immunotherapy analysis

We conducted a thorough analysis using the Genomics of Drug Sensitivity in Cancer (GDSC) database (https://www.cancerrxgene.org/) [PMID: 23180760] to identify drugs that exhibited a significant correlation (p-value ≤ 0.05) with m5CSS scores. Subsequently, we compared the sensitivity variations of these drugs across different subgroups. Next, TIDE was implemented to validate the scoring system in predicting immunotherapy response and to reconcile the disparities in immunotherapy predictive indicators, such as TMB, HRD, and TIDE between the m5C score groups.

### RT-qPCR analysis

The experiments were authorized by the Ethics Committee, and RT-qPCR analysis was described in earlier publications [31]. Using the TRIzol reagent (Invitrogen), total RNA was isolated from Peripheral Blood Mononuclear Cell from 16 AML patients and 10 healthy individuals. By cDNA synthesis kit (Takara), total RNA was reverse transcribed into cDNA with oligo (dT) primers. TB Green Premix Ex Taq II (Takara) was adapted for RT-qPCR following the recommendations of manufacturers. Using the comparative CT (2-ΔΔCT) approach and adjusting for B-actin levels, the relative quantification was carried out. Supplementary Material Table 2 contains a list of the RT-qPCR primer sequences.

**Table 2:** The RT–qPCR primer sequences used in the study.

**Genes**	**Primer sequences**
β-actin	F: TGGCACCCAGCACAATGAA
	R: TAAGTCATAGTCCGCCTAGAAG
LPO	F: GGGACTACCTACCCATTTTGC
	R: CCAGGCGGAACATACTAGAGG
CLIP4	F: CCTGGGAGCAGACATTAGTTTG
	R: GCACACAAGTTGTATGCTGCAA
PLXNC1	F: AGAGTCCAACCAATCGCATCA
	R: AGTCCTGTTCATTACCACGGT
CPNE8	F: GGGCAGTCACAATTCAACGTA
	R: TTGCGTCCCTCCCTTAATGTA
BCL2A1	F: TACAGGCTGGCTCAGGACTAT
	R: CGCAACATTTTGTAGCACTCTG
MPO	F: TGCTGCCCTTTGACAACCTG
	R: TGCTCCCGAAGTAAGAGGGT
PRDM16	F: CGAGGCCCCTGTCTACATTC
	R: GCTCCCATCCGAAGTCTGTC
LSP1	F: GGAGCACCAGAAATGTCAGCA
	R: TCGGTCCTGTCGATGAGTTTG
SIX3	F: CTGCCCACCCTCAACTTCTC
	R: GCAGGATCGACTCGTGTTTGT
ACSM1	F: GGGGCATCCACAAATCCTTC
	R: TCTTGGGGCTCCAAATTCTGA
CFD	F: GACACCATCGACCACGACC
	R: GCCACGTCGCAGAGAGTTC
HTR1F	F: ACTTGACCTCAGAGGAACTGT
	R: ATTGCAGCGATCACAAGGGAG
SORT1	F: GGGGACACATGGAGCATGG
	R: GAATAGACAATGCCTCGATCAT

### Statistical analysis

To determine the statistical significance of three or more groups, the Kruskal-Wallis test was utilized, and the Wilcoxon test was used to examine if tumor samples from various clinical groupings showed different patterns of expression. By using Spearman correlation analysis, the correlation coefficients were determined. The R package survminer determined the cutoff for each dataset subgroup based on the correlation between the m5C score and the survival of patients. The R package maftools was employed to display the mutation status of all genes as well as the level of mutation in m5C-related genes. Similarly, the CNV of m5C-related genes in the overall tumor sample was characterized and their specific distribution on chromatin was visualized using the R package Circos. Interactions among m5C-related genes were resolved using the String database (https://cn.string-db.org), and those with medium confidence (Score>0.4) were assessed. The significance level for each two-sided test was p<0.05.

### Data availability

The datasets analyzed for this study can be found in the UCSC Xena database (https://xenabrowser.net/datapages/), TCGA (https://portal.gdc.cancer.gov/) and the GEO database (https://www.ncbi.nlm.nih.gov/geo/).

## RESULTS

### Expression, mutation and copy number variation of m5C gene

**Figure 1:**
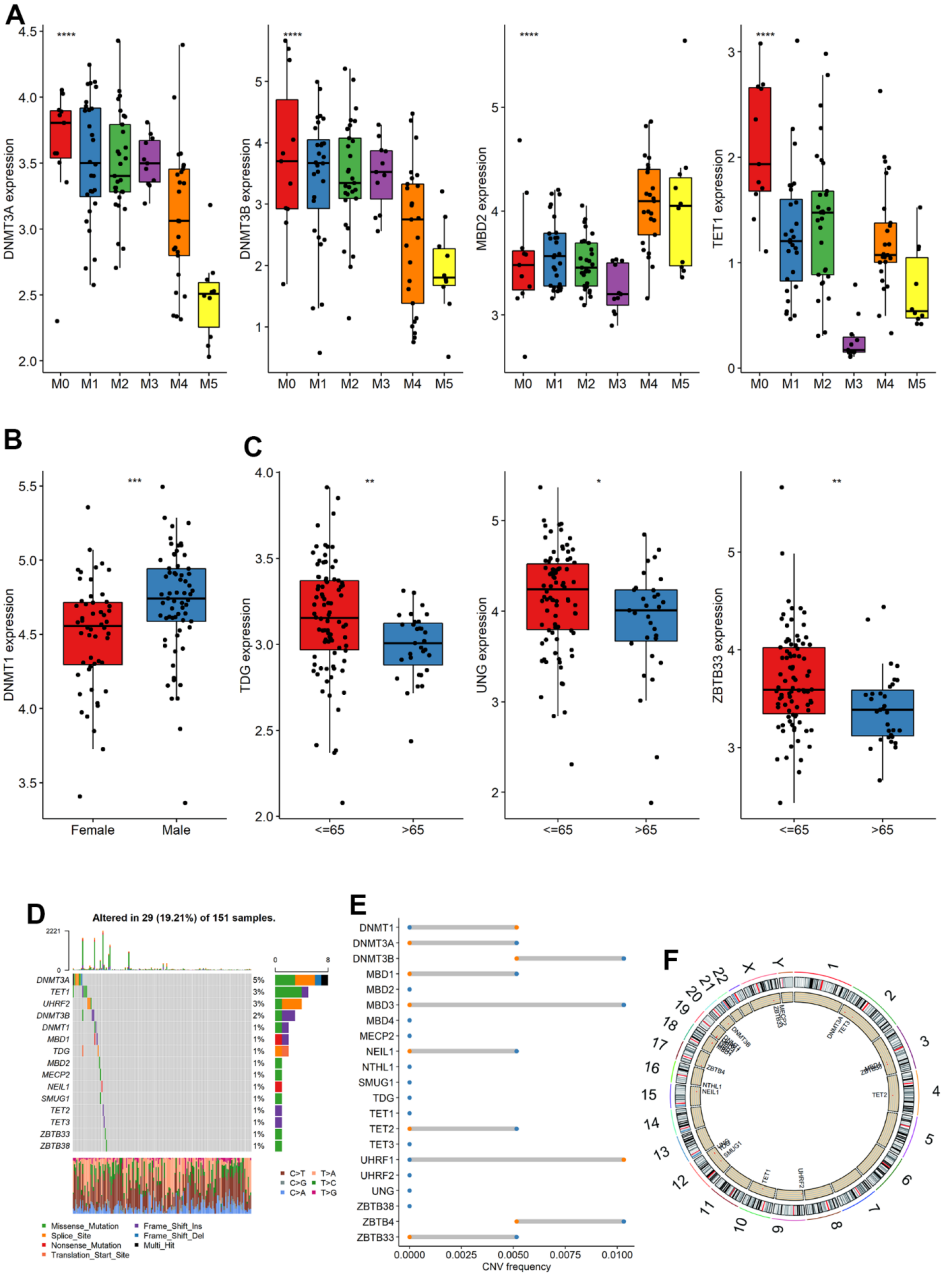
**Expression, mutation and copy number variation of m5C gene.** (**A**–**C**) The expression differences of m5C gene among different groups (FAB, Sex, Age), (**D**) mutation, (**E**) copy number variation and (**F**) localization of m5C gene on chromosome.

The analysis of m5C gene expression differences among subgroups based on clinical characteristics revealed several significant findings. Firstly, when considering the French-American-British (FAB) typing groups, we observed significant differences in the expression of DNMT3A, DNMT3B, MBD2 and TET1 (Figure 1A). Additionally, DNMT1 exhibited significant differences between gender groups (Figure 1B). Furthermore, three genes, namely TDG, UNG, and ZBTB33, showed significant differences among age groups (Figure 1C), respectively. The study summarized the mutations in the m5C gene of the TCGA dataset. 29 of the 151 samples underwent m5C regulation (19.21%), and followed by TET1 and UHRF2, the DNMT3A gene displayed the highest mutation frequency, both with a mutation rate of 3% (Figure 1D). CNV alteration frequency displayed that 21 regulators were partially CNV altered and some copy number amplification deletion occurred, while DNMT1 and UHRF1 had extensive CNV deletion frequency (Figure 1E). Figure 1F depicted where the CNV change occurred. To analyze whether the m5C gene was associated with OS, the median gene expression values were used to divide the samples into high and low expression groups, and univariable cox regression analysis showed that DNMT3A and ZBTB38 were substantially linked to OS (Supplementary Figure 1A, 1B). The aforementioned research revealed significant heterogeneity in the landscape of genetic and expression alterations, suggesting the crucial role that m5C control of expression disequilibrium plays in the initiation and development of AML.

### Cluster analysis of m5C regulators

**Figure 2:**
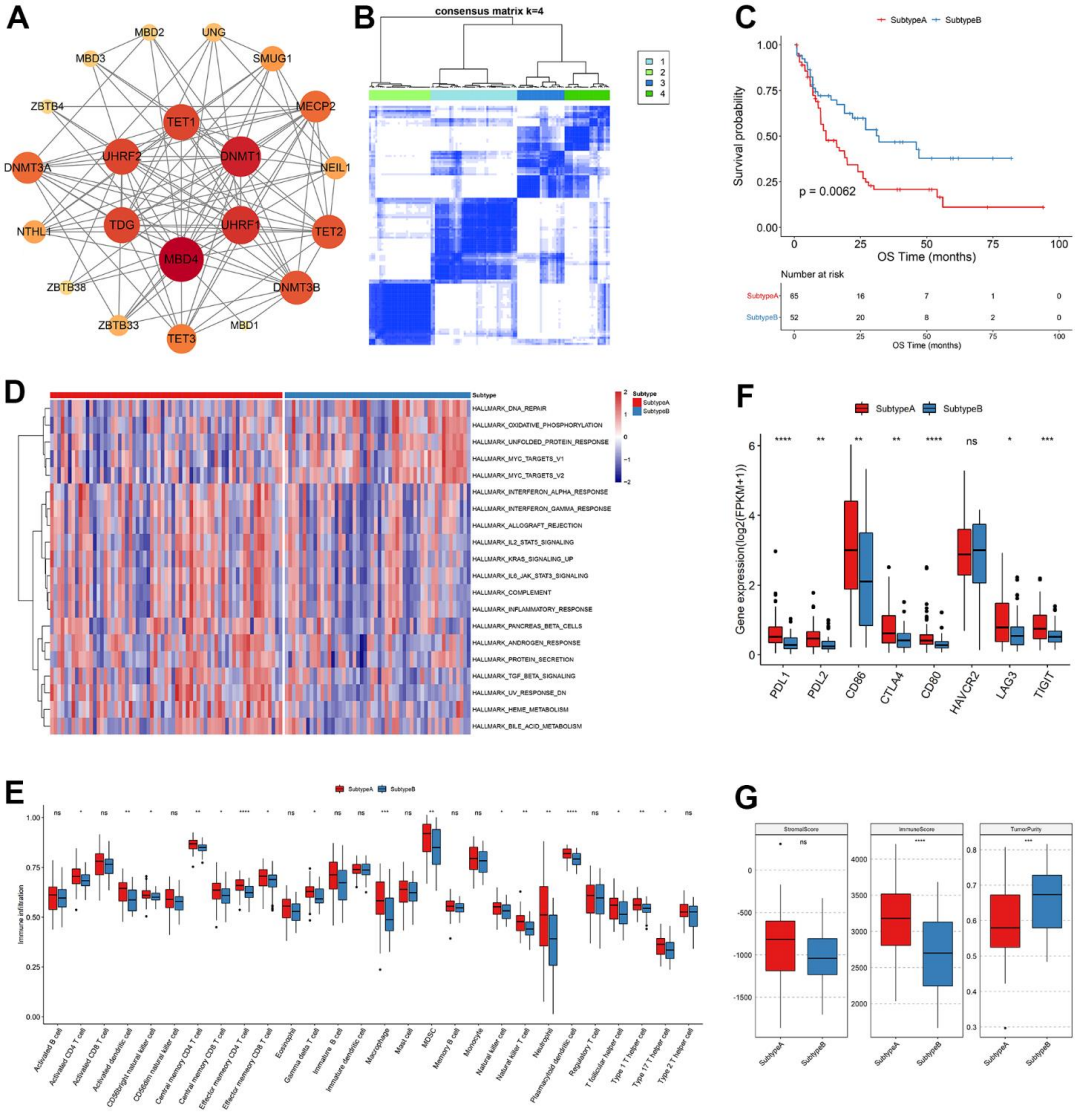
**Clustering analysis of m5C regulators.** (**A**) Interaction relationship plot, (**B**) sample correlation matrix of consistent clustering, (**C**) KM curves of new subtypes Subtype A, Subtype B, OS and (**D**) GSVA analysis, (**E**) differences in immune cell abundance between subtypes, (**F**) differences in immune checkpoint gene expression and (**G**) differences in stromal score, immune score and tumor purity.

Analysis of interactions of high-confidence m5C genes using the String database indicated that m5C genes had more extensive connections (Figure 2A), and DNMT1, DNMT3A, DNMT3B, and other genes were closely bound, suggesting that they may be hub genes for m5C RNA methylation regulators. Subsequently, consistent clustering regarding the expression profile of the m5C gene identified four subgroups with significant differences in OS (Figure 2B) which were named Subtype 1-4. Principal component analysis (PCA) demonstrated a clear differentiation among the four subgroups (Supplementary Figure 1C). The heatmap of m5C genes exhibited distinct and noticeable differences among the four subtypes. ZBTB4 expression of M7 patients in Subtype1 was highly expressed (Supplementary Figure 1D), and survival analysis revealed that these four subtypes exhibited substantial differences. Subtype 3 has the worst survival among them (Supplementary Figure 1E). Additionally, Subtype1, Subtype 3 were merged into Subtype A while Subtype2, Subtype4 were merged into Subtype B according to their OS outcomes. Subtype B has a significant better survival (Figure 2C).

To examine the biological distinctions among the sample groups, we conducted GSVA, resulting in the identification of 20 significantly different pathways between the two groups. Notably, Subtype A and Subtype B exhibited a strong association with heme metabolism, the MYC gene set and other pathways. The m5C genes downregulated in various pathways that are found to be related to AML in previous literature, such as interferon response [PMID: 36964168], protein secretion [PMID: 36475901], bile acid metabolism [PMID: 36084349]. Specifically, Subtype A displayed enrichment in metabolic pathways involved in heme metabolism and erythropoietic differentiation, indicating a potential role in promoting these processes. Conversely, Subtype B was primarily characterized by an inhibition of heme metabolism, suggesting a potential disruption or suppression of these metabolic pathways. Heme metabolism plays a crucial role in multiple biological processes, including oxygen transport, cellular respiration, and enzymatic reactions. It is intricately linked to erythropoiesis, the process of red blood cell production. The dysregulation of heme metabolism in Subtype B might result in impaired erythropoietic differentiation and altered cellular responses to heme-related stimuli. These findings shed light on the distinct molecular characteristics and functional differences between Subtype A and Subtype B in relation to heme metabolism. The identification of these pathways provides valuable insights into the underlying mechanisms driving these subtypes and may have implications for further research and targeted interventions in acute myeloid leukemia (Figure 2D). When the immune cell abundance of the tumor samples was calculated for TME cell infiltration using ssGSEA, it was shown that the bulk of the immune cell abundances was significantly distinct between the two subtypes (Figure 2E). Infiltrating innate immune cells in m5C Subtype A included plasmacytoid dendritic cells, mast cells, MDSC, natural killer cells, macrophages, and eosinophils. Comparably, it was examined if the two subtypes of immune checkpoint gene expression significantly differed from each another. PDL1, PDL2, CD86, CTLA4, CD80, LAG3, and TIGIT genes were significantly expressed differently between the Subtype A and Subtype B groups, while HAVCR2 genes were not (Figure 2F). Finally, the immunological score and tumor purity were discovered to be significantly different by analyzing the distribution of the immune score, stromal score, and tumor purity between the two subtypes of samples (Figure 2G).

### Patterns of m5C methylation modification mediated by m5C regulators

**Figure 3:**
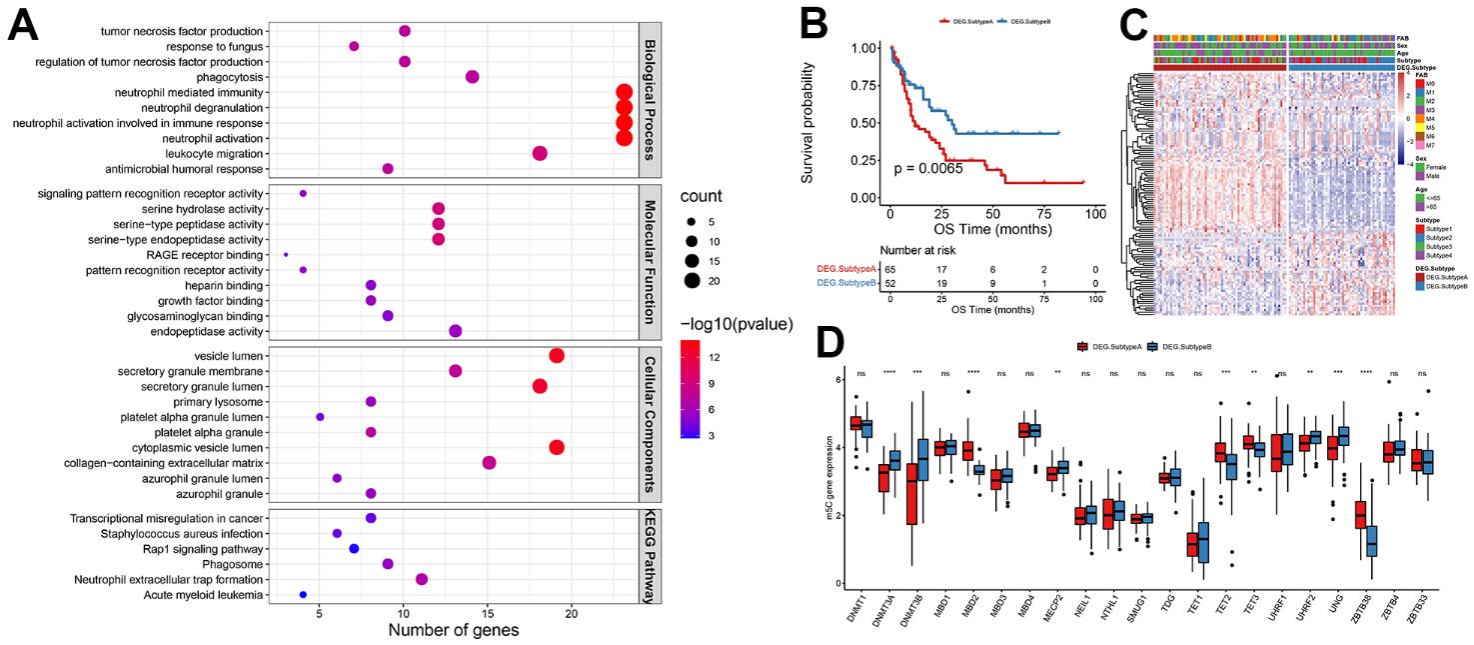
**Patterns of m5C methylation modification mediated by m5C regulators.** (**A**) Functional enrichment analysis of differential genes, (**B**) KM curves of OS, (**C**) heat map of m5C gene expression distribution, (**D**) and histogram of m5C gene expression distribution.

114 differential genes encoding proteins were identified from Subtype A and Subtype B after further investigation into the likely biological behavior of each m5C alteration pattern (threshold |log2FC|>1 and padj<0.05). According to the findings presented in Figure 3A, the top 10 significant pathways in terms of biological processes (BP), molecular functions (MF), cellular components (CC), and Kyoto Encyclopedia of Genes and Genomes (KEGG) were predominantly associated with the regulation of tumor necrosis factor (TNF) production. This demonstrated that immune control in the TME is significantly influenced by m5C alterations. These differential genes were largely enriched in biological functions associated with the immune system, such as regulation of tumor necrosis factor production, neutrophil activation involved in immune response, and leukocyte migration. To further validate this regulatory mechanism, the tumor samples were subsequently clustered consistently based on the differential gene expression levels. The samples were categorized into two classes with expression patterns, DEG. Subtype A, and DEG. Subtype B. This suggested that m5C methylation modification patterns were indeed present in AML. Significant OS differences between these two subtypes, as determined by differential gene expression, were shown by survival analyses, with 52 of 117 AML patients clustered in DEG. Subtype B associated with a better prognosis, and patients with DEG. Subtype A (65 patients) with a worse prognosis (Figure 3B). The analysis also identified that different signature genes were found in two separate gene clusters, with opposite DEG. Subtype A and DEG. Subtype B m5C patterns observed for the FAB classification. FAB classes M4 and M5 concentrated in DEG. Subtype A and M2 patients were clustered in DEG. Subtype B (Figure 3C). Significant changes in m5C regulator expression were found in m5C DEG. Subtype A and DEG. The results of Subtype B were in line with what was expected given the m5C methylation modification pattern (Figure 3D).

### Gene signature construction by m5CSS

**Figure 4:**
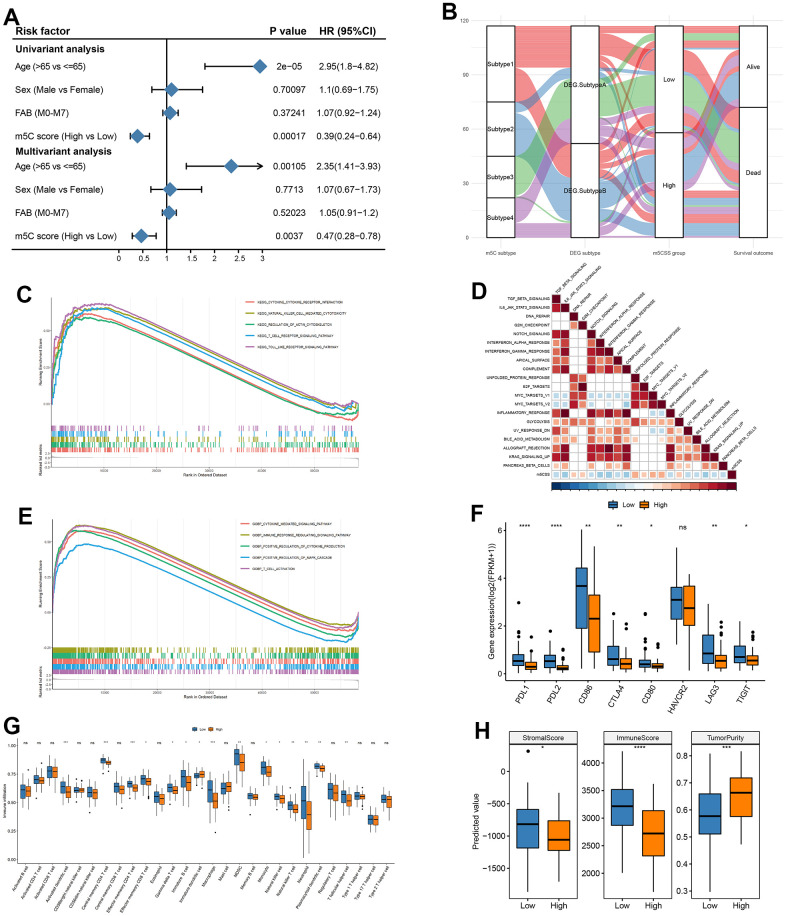
**Construction of m5C gene signature.** (**A**) Univariate cox analysis in training set and (**B**) impact plots (from left to right, m5C isoforms, differential gene expression subtypes and m5C score classification and final sample survival, respectively), (**C**) KEGG enrichment for high and low scoring subgroups, (**D**) correlation between m5C score and hallmark pathway, (**E**) GO enrichment for high and low scoring subgroups, (**F**) immune checkpoint gene expression differences, (**G**) immune cell differences, (**H**) stromal score, immune score and tumor purity in high and low subgroups.

Next, we investigated the value of m5CSS as a prognostic predictor was investigated in AML. The univariable cox regression model with a cutoff of P<0.005 was used to recognize 13 OS-related differential genes to identify genes significantly correlated with OS as characteristic genes. The m5C score was generated in the training and validation sets using the m5CSS formula. The m5CSS formula refers to the sum of PC1 and PC2 values for each sample in PCA analysis. The samples were then separated into high and low subgroups based on the median m5C score (Supplementary Figure 2A). Their survival distribution in Supplementary Figure 2B reflected that patients with high m5C scores have a better outcome. The m5C group with a high score displayed increased SIX3 gene expression in the training set (Supplementary Figure 2C). Significant OS differences between the high and low subgroups were discovered using survival analysis (Supplementary Figure 2D). These finding was also validated in the validation dataset (GSE37642) (Supplementary Figure 2E–2H). The m5CSS was evaluated in the training set and validation set by combined univariate cox and multifactor cox regression (including patient age, gender, and FAB classification) analyses, respectively. Age and m5C score were strongly connected with OS in the univariate and multifactor cox regression analysis, showing that the m5C score can indeed be employed as an independent prognostic indicator in the training set (Figure 4A). Likewise, in the validation set, when univariable cox regression analysis used, only the m5C score was significantly associated with OS, and the further results of the multifactorial analysis revealed that the m5C score was remained strongly related to OS, suggesting the role of m5C score as a predictor (Supplementary Figure 3A). To clearly describe the information about the different categories of the samples, the subtypes to which the samples belonged and their corresponding m5CSS groupings and prognosis were depicted with a shocking plot (Figure 4B), which showed that m5C DEG. Subtype B had and high m5CSS and the lowest survival rate. Then, Wilcoxon-test was used to analyze whether there were distinctions in m5CSS between different m5C subtypes and DEG subtypes, and the results illustrated that the m5C score was markedly different between subtypes of both of these classifications (Supplementary Figure 3B, 3C). In addition, the m5C scores of tumor samples in FAB subgroups were different, even though age and sex clinical characteristics subgroups did not differ significantly, (Supplementary Figure 3D–3F).

Based on MSigDB cancer hallmarks, the Pearson correlation between the m5C score and cancer hallmarks was calculated to evaluate their correlation. The m5C score was discovered to have a substantial inverse relationship with a number of pathways, including INTERFERON _GAMMA RESPONSE and MYC TARGETS V2 (Figure 4D). Similarly, parsing the GO and KEGG enrichment of m5CSS samples with high and lows scores revealed that biological processes include the MAPK cascade, cytokine-mediated signaling pathway, and T cell activation that are positively regulated by high scoring and highly expressed genes (Figure 4C, 4E). The m5C score improved evaluation of the m5C modification pattern of specific tumors and provided additional evaluation of the tumor TME cell infiltration characteristics. Subsequently, combining the immune cell abundance of the disease samples calculated by the three methods of CIBERSORT, xCELL, and xxGSEA, most immune cells were considerably different in abundance between the two score groups (Figure 4G). Additionally, to determine whether there were any appreciable variations between the high and low score categories, the expression of immune checkpoint genes was evaluated. The bulk of the low score groupings were found to have significantly higher gene expression (Figure 4F). Finally, in both the high and low scoring groups, there were substantial disparities in the distribution of the stromal score, immunological score, and tumor purity discrepancies (Figure 4H).

**Figure 5:**
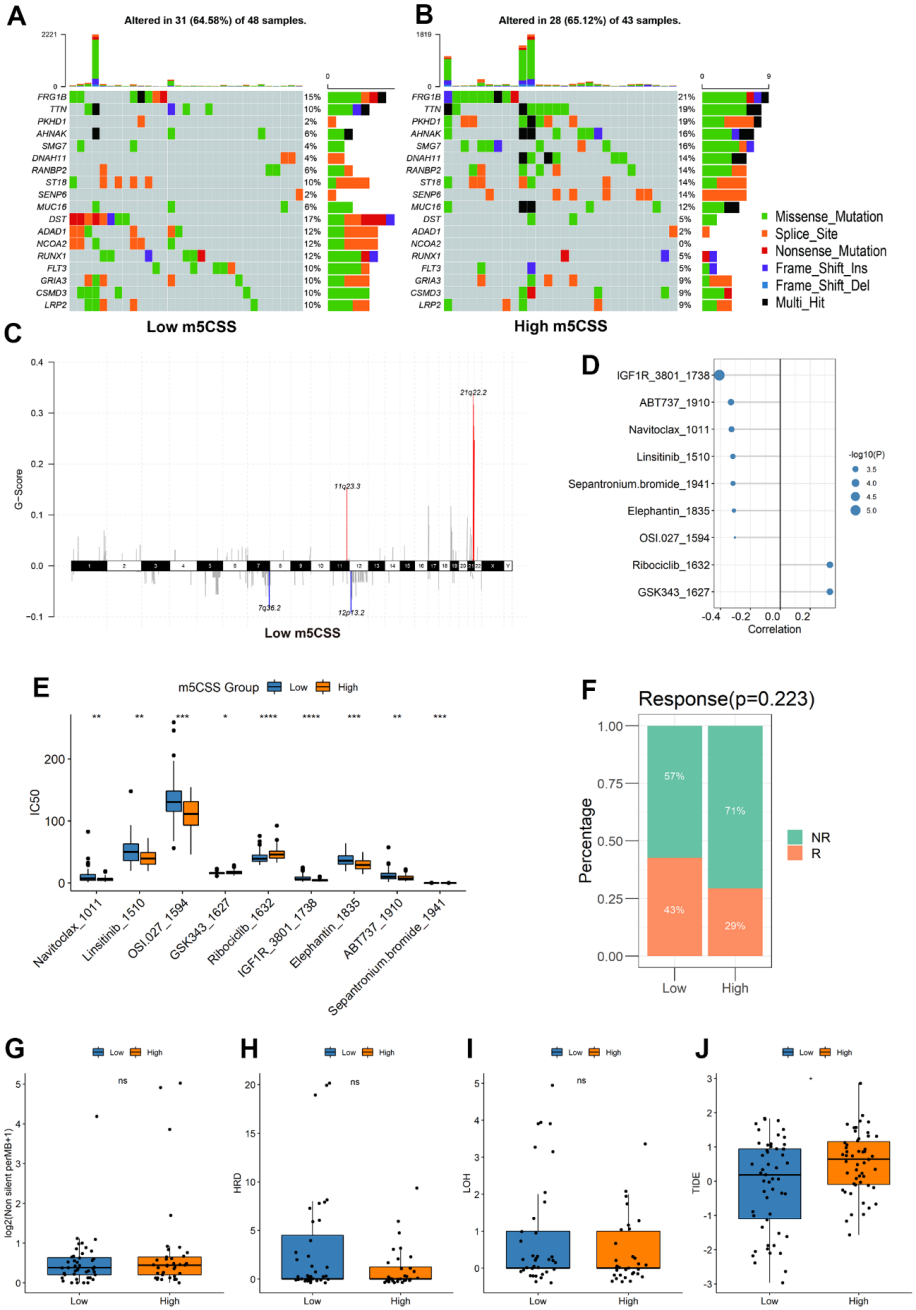
**Distribution of m5C score in clinical characteristics samples and m5C subtypes.** (**A**, **B**) Differences in mutations between high and low m5C score groups, (**C**) differences in copy number variants, (**D**) correlation between m5C score and drugs, (**E**) differences in drug resistance between high and low score groups, (**F**) proportion of patients responding to immunotherapy, differences in (**G**) TMB, (**H**) HRD, (**I**) LOH, (**J**) TIDE between high and low score groups.

Afterward, we examined the variations in the distribution of somatic mutations between the two m5C scores. While PKHD1 had 2% mutations in the low m5C score group compared to while only 19% in the high m5C score group, TTN showed lesser mutations in the low m5C score group at 10% compared to samples from the high m5C score group at 19%. The Fisher-test was utilized to screen for genes with mutational differences between the two categories at P<0.05 to further characterize the mutational differences between the two groups, and ultimately no genes were discovered to be substantially different (Figure 5A). The GISTIC2 tool on the GenePattern website was next used to examine the CNV data from the high and low-scoring group samples for their amplification deletions in an attempt to find statistically different levels of amplification deletions, however, no such differences were discovered (Figure 5B). All in all, these findings will open up new avenues for investigation into the mechanisms behind TME development, immune checkpoint blockade therapy, and m5C methylation alteration in tumor somatic mutations.

### Drug resistance analysis of m5C groups with high and low scores

To predict drug resistance in the samples from the subgroups with high and low score, the GDSC dataset was employed. The Pearson correlation between the m5C score and drug IC50 was then determined. Most medications demonstrated a greater correlation with the m5C score (Figure 5D, |cor|>0.3, p < 0.05). It was demonstrated in Figure 5E that between the high and low score groups, there were significant disparities in each type of treatment resistance. The results of IGF1R_3801_1738 and Ribociclib_1632 demonstrated a substantial difference between the high and low groups, thus demonstrating the value and potency of the m5C score as a predictor for prognosis and therapeutic response assessment of immunotherapy.

### Evaluation of immunotherapy efficacy in m5C groups with high and low scores

The m5CSS traits established in the AML cohort were applied to other independent cohorts to further test the stability of the system. With the assistance of TMB and HRD, the TIDE was used to predict how the sample would respond to immunotherapy. The results suggested that low m5C scores were more responsive to immunotherapy (Figure 5F), and between the groups with high and low scores, there was a significant statistical difference in the TIDE scores (Figure 5G–5J).

### Verification of prognostic gene expression by RT-qPCR

**Figure 6:**
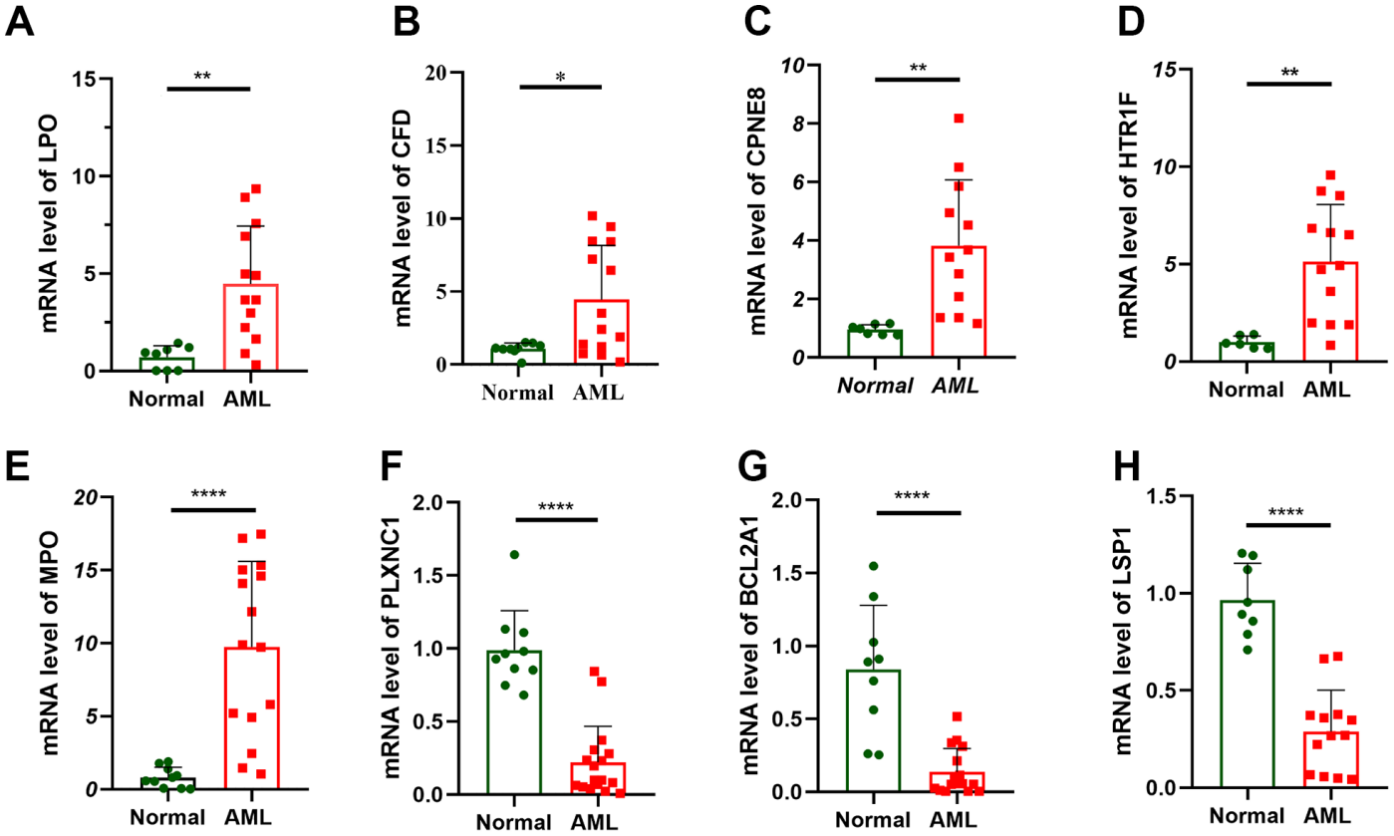
Verification of prognostic gene expression by RT-qPCR. RT-qPCR analyses of LPO (**A**), CFD (**B**), CPNE8 (**C**), HTR1F (**D**), MPO (**E**), PLXNC1 (**F**), BCL2A1 (**G**) and LSP1 (**H**) expression in 16 AML patient and 10 healthy people.

Finally, 13 hallmark prognostic genes were examined for expression levels using RT-qPCR in 16 AML patients and 10 healthy people from The Central People’s Hospital of Zhanjiang. Among these 13 genes, the expression levels of PRDM16, SIX3, CLIP4, SORT17 and ACSMl between normal and AML samples did not significantly alter. LPO, CFD, CPNE8, HTR1F, and MPO showed noticeably higher expression in the AML sample than in the normal sample, while LSPl, PLXNCI and BCL2A1 showed decreased expression (Figure 6). The level of expression showed a substantial statistical difference, indicating that these 8 genes were risk factors of and could be potential treatment targets for AML patient.

## DISCUSSION

One of the most prevalent and deadly hematological malignancies is AML, which prevents myeloid differentiation to form leukemic stem cells that may self-renew (LSC) [17]. Enhancing early detection is crucial to treat AML. Despite recent advancements in combination therapies, the diagnosis and prognosis of AML are still not satisfactory. Furthermore, a major obstacle is the absence of confirmed biomarkers for early diagnosis, and the existing clinical markers lack precision and specificity in predicting patient prognosis and treatment response. Therefore, it is imperative to investigate the genetic and epigenetic mechanisms underlying AML development to identify new treatment targets and biomarkers. While RNA alterations have been associated with cancer growth and disease pathogenesis, the potential connection between AML and m5C (5-methylcytosine) remains unknown. To address this gap, we conducted a study using gene expression profiles of 117 AML patients obtained from the TCGA (The Cancer Genome Atlas) database. From these profiles, we developed an m5C-related scoring system. To the best of our knowledge, this is the first comprehensive investigation into the role of m5C in AML. By studying m5C and its impact on AML, we aim to uncover new insights into the disease’s mechanisms and potentially identify novel biomarkers for early detection. This research may contribute to the development of more precise diagnostic tools and targeted therapies, ultimately improving patient outcomes in AML.

M5C is frequently present in long non-coding RNAs and participates in several biological processes related to cancer and growth. Current findings have demonstrated that m5C-related genes can control the breast cancer tumor immunological microenvironment [32]. By increasing m5C levels, NSUN2 encourages gastric cancer (GC) cells to proliferate, migrate, and invade [33]. In bladder cancer (BLC), m5C modification of PKM2 mRNA enhances glucose metabolism [34]. Despite extensive studies, the whole regulatory function of m5C remains unclear, and further studies of m5C-related regulators are necessary to elucidate their potential regulatory mechanisms in TME.

In the TCGA-LAML dataset, we examined the m5C gene expression patterns in AML and extensively described their expression. After confirming the differences of m5C genes expression levels among clinical factors such survival, FAB subtyping, gender, age and mutation. We performed unsupervised clustering to identify four subtypes which shows distinct differences in survival and expression levels. The four subtypes were combined into two subtypes according to their survival. Subsequently, the immune cell abundance of tumor samples was calculated using algorithms of three tools, CIBERSORT, xCELL, and ssGSEA, respectively, to evaluate how immune cells were distributed among the various sample subgroups. The process of heme metabolism can indeed have divergent outcomes between the two subtypes. Clinical parameters, immune infiltration levels, immunological characteristics, immune checkpoint medication responsiveness, and immunotherapy effectiveness were all examined for associations with m5C. The findings demonstrated that the immune cell infiltration characteristics and patient prognosis were both correlated with the m5C gene. Based on this, m5CSS was constructed. We discovered that the model performed similarly well in independent data. With considerable OS differences, the score might classify the samples into high and low groups. The m5C score can act as an independent prognostic indicator to more precisely assess the TME cell infiltration features of tumors and measure the pattern of m5C modification in individual tumors.

Additionally, the study revealed a substantial inverse connection between the m5C score and the INTERFERON_GAMMA_RESPONSE and MYC_TARGETS_V2, and that IFN gamma production is a hallmark of innate and adaptive immunity [35]. The excessive release of IFN gamma has been connected to the chronic inflammatory disorders and etiology of autoimmune, in addition to its crucial role in host defense. And MYC is a human gene that regulates the promotion of cell growth and proliferation [36]. Then, analysis of medication resistance and immunotherapy response revealed that immunotherapy was more responsive to low m5C scores. The study discussed support the value of the m5C score in predicting immunotherapy and imply that m5C alteration may be an important factor of immunotherapy response. However, in terms of the validation of our experiments, we conducted RT-qPCR and successfully confirmed our research findings. To further develop and apply m5CSS, our future work will consider conducting additional experiments such as immunohistochemistry or Western blot for in-depth validation of the results.

To summarize, the m5C score can be applied in clinical practice to thoroughly evaluate the m5C methylation modification pattern and its associated TME cell infiltration characterization in specific patients, which will further define the immunophenotype of the tumor, and help direct more efficient clinical management. We subsequently demonstrated that the m5C score can indeed be utilized as a prognostic indicator to evaluate the clinicopathological parameters of patients and predict patient survival independently. Drug resistance and the clinical outcome of immunotherapy can both be forecasted with the m5C score.

## CONCLUSIONS

In this study, we determined how m5C alteration patterns in AML function. Based on the m5C profiles with different modification patterns, m5CSS with good prognostic efficacy was constructed. Interestingly, the m5C score was able to quantify m5C subtypes, and the score might be applied as an independent prognostic indicator for prediction of individual responses to immunotherapy. Our thorough examination of m5C modifications may offer fresh perspectives into the investigation of AML and potentially aid in the future creation of TME and immunotherapies.

## Supplementary Material

Supplementary Figures
