# Supplementary material for: m5C methylation modification guides the prognostic value and immune landscapes in acute myeloid leukemia
Source: Aging (Albany NY). 2023 Sep 25;15(18):9858–76. doi: 10.18632/aging.205059 (PMC10564437; doi:10.18632/aging.205059)
Supplement: Supplementary Figures [file aging-15-205059-s001.pdf]

SUPPLEMENTARY FIGURES

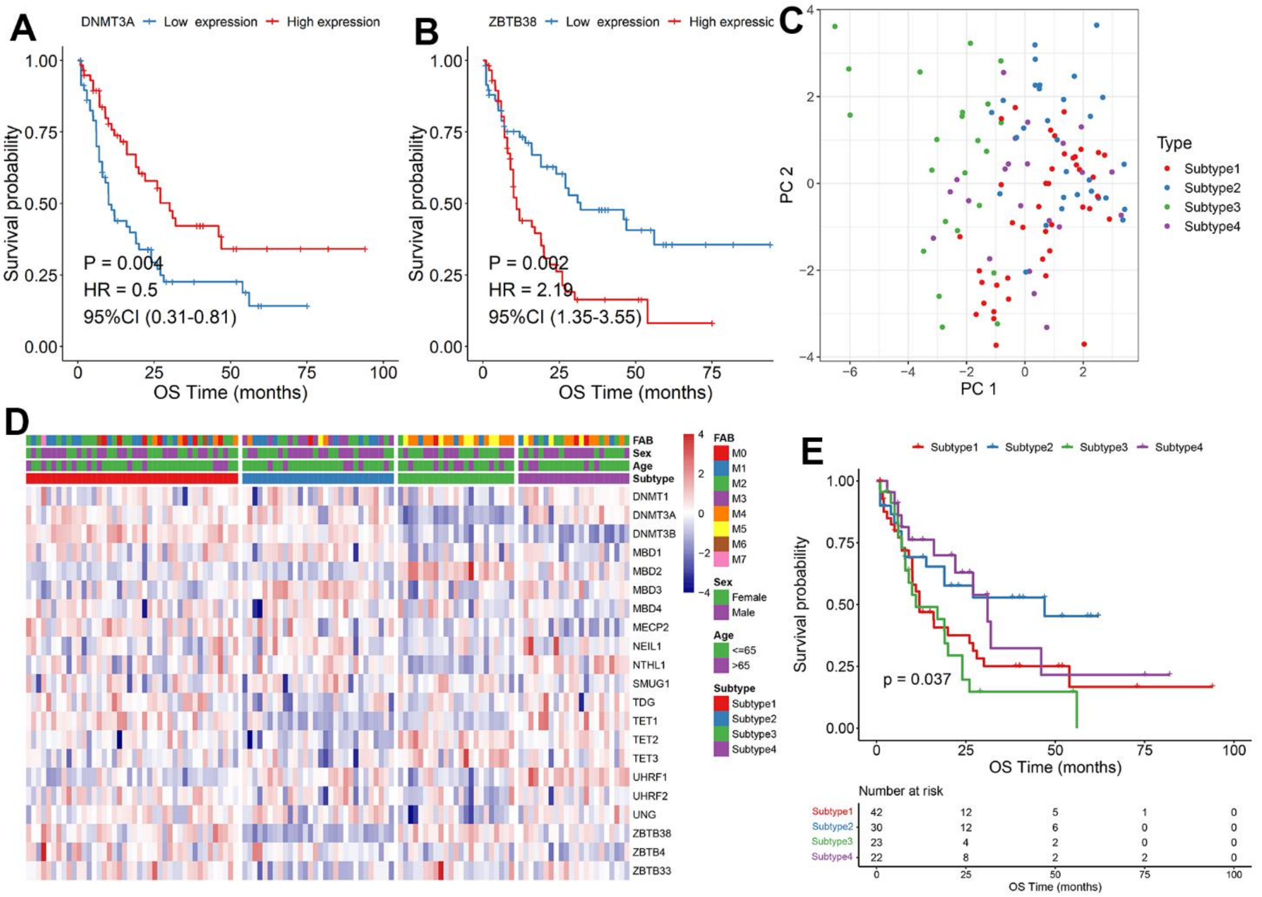

Supplementary Figure 1. m5C gene prognostic correlation and clustering analysis. (**A**) KM curves of DNMT3A.and (**B**) ZBTB38, (**C**) principal component analysis of Subtype 1-4, (**D**) m5C gene expression distribution in Subtype 1-4, (**E**) KM curves of Subtype 1-4.

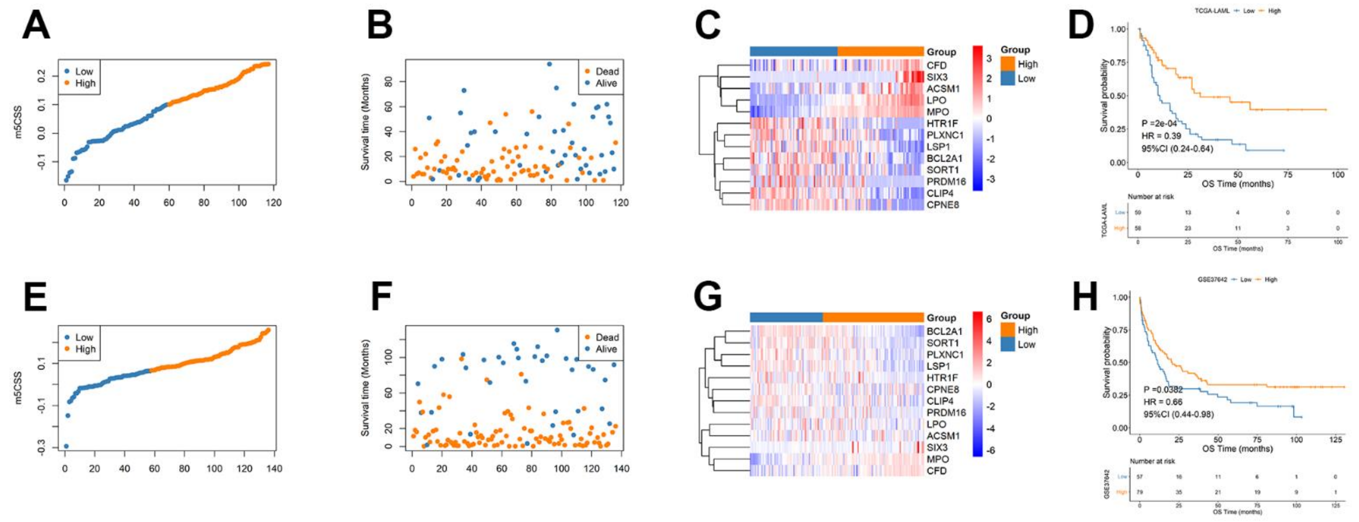

Supplementary Figure 2. The m5CSS gene characteristics of training and validation set. In training set, (**A**) the m5CSS distribution, (**B**) survival time of different samples, (**C**) expression of model genes, (**D**) survival analysis of high and low m5CSS group; in validation set, (**E**) m5CSS distribution, (**F**) survival time of different samples, (**G**) expression of model genes, (**H**) survival analysis of high and low m5CSS group.

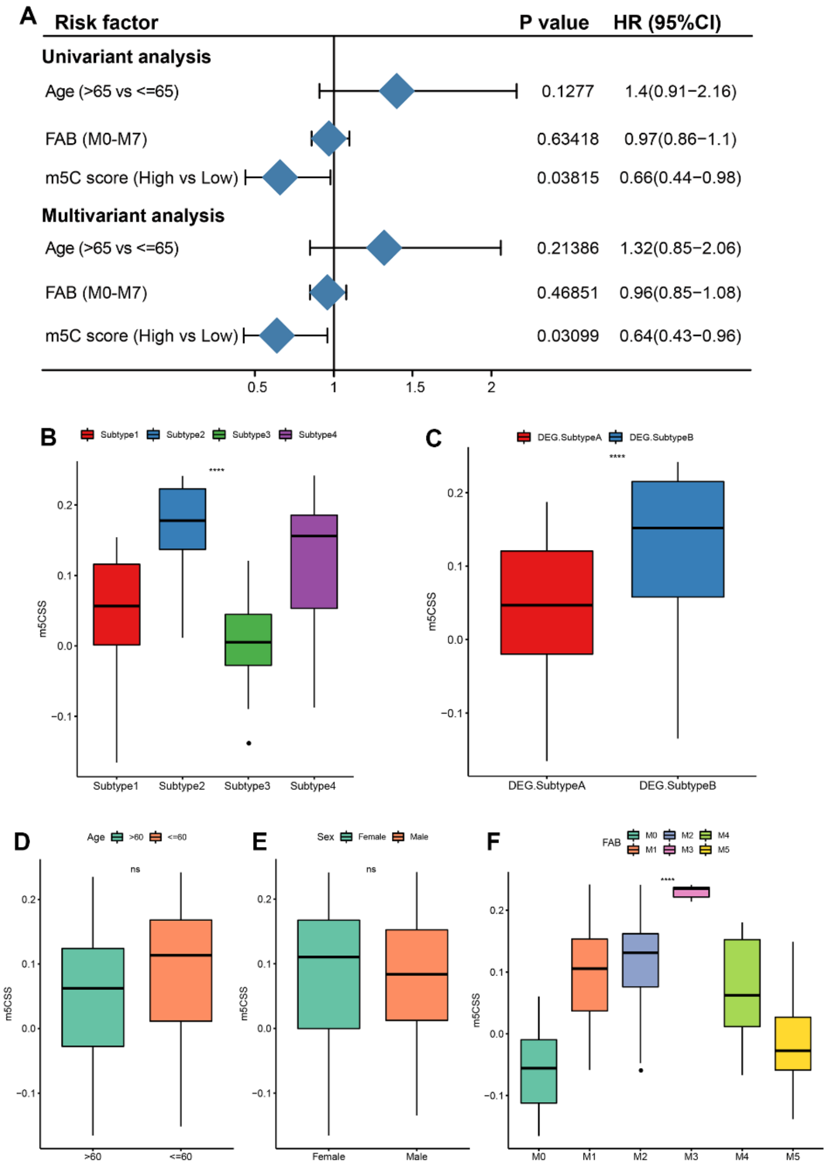

Supplementary Figure 3. Distribution of m5C score for clinical characteristics samples and m5C subtypes. (**A**) Univariable cox analysis in validation set, (**B**) m5C score difference between m5C subtypes, (**C**) m5C score difference between DEG subtypes, (**D**–**F**) m5C score distribution of different clinical characteristics samples (Age, Sex and FAB, respectively).
